# Consumption of snacks and dental caries among Finnish young men: a cross-sectional epidemiological study

**DOI:** 10.1007/s10266-019-00473-z

**Published:** 2019-11-12

**Authors:** Tarja Tanner, Laura Harju, Jari Päkkilä, Pertti Patinen, Leo Tjäderhane, Vuokko Anttonen

**Affiliations:** 1 Department of Cariology, Endodontology and Pediatric Dentistry, Research Unit of Oral Health Sciences, University of Oulu, P.O. Box 5281, 90014 Oulu, Finland; 2 Department of Mathematical Sciences, University of Oulu, P.O. Box 8000, 90014 Oulu, Finland; 3 Centre for Military Medicine, Finnish Defence Forces, P.O. Box 5, 11311 Riihimäki, Finland; 4 Department of Oral and Maxillofacial Diseases, University of Helsinki, Helsinki University Hospital, P.O. Box 41, 00014 Helsinki, Finland; 5 Medical Research Unit, Oulu University Hospital and University of Oulu, P.O. Box 5281, 90014 Oulu, Finland

**Keywords:** Dental caries, Diet counselling, Health behaviour, Physical exercise, Snacking

## Abstract

The aim of this study was to investigate the frequency of consumption of snack products, as well as the association between snacking and restorative treatment need, and associated factors among a healthy Finnish male population. Approximately 8500 conscripts answered a computer-based questionnaire covering their snacking habits and other health behaviours. Restorative treatment need and history (DT, DMFT) were examined by trained and calibrated dentists. Cross-tabulations were used to investigate the associations between snacking habits and the other researched variables, and logistic regression analyses (odds ratio and 95% confidence interval) were used to investigate the variables influencing the restorative treatment need. In the present study, almost one-third of the study group consumed snack products daily, most often fizzy and energy drinks. Only 10% had received a diet counselling. The most common situations involving snacking were at the cinema and while playing computer games. According to Pearson’s Chi square test, snacking was associated with smoking and snuffing and infrequent tooth brushing (*p* < 0.001). According to the regression analyses, daily snacking, smoking, and doing exercise daily increased the odds for restorative treatment need whereas higher education level and tooth brushing twice or more often per day decreased the odds for restorative treatment need. It can be concluded that daily snacking is common among Finnish young men and is associated with restorative treatment need. Snacking is also associated with other harmful oral and general health habits. Individual dietary counselling should be routinely offered to everybody in dental clinics.

## Introduction

Over the past 15 years, the sales of chocolate and sweets have increased in Finland, although at the same time the sales of sugar have remained steady. In 2005, the total import of chocolate and sweets were approximately 135MEur, while in 2016, the respective figure was 221 MEur [1].

Especially adolescents and young adults snack several times daily [2, 3]. Typical snacks in Finland include sweets, fizzy drinks, crisps, and light meals (such as bread, porridge, muesli) [4]. In recent decades, an increasing proportion of daily energy intake has been received from snacks. Hoppu et al. [2] have reported that up to 41% of daily energy intake of Finnish adolescents originates from sugary snacks and drinks, which is more than from any daily meal. Sugar-sweetened juice, soft drinks, and sweets are the most common sources of sucrose among Finnish adults [5]. The frequency of the intake of free sugars or sugar in food plays a significant role in the development of dental caries lesions [6]. Recently, the same has also been shown to be true for the total sugar intake [7, 8], especially when combined with poor oral hygiene. Recent literature on dietary patterns, including snacking in association with oral health, is scarce.

Demineralisation of enamel and dentine is caused by bacteria in dental plaque [9]. Oral bacteria can ferment all common mono- and disaccharides, together with hydrolysed polysaccharide starch. Therefore, these carbohydrates are potentially cariogenic [10]. Even though the amount of sugar in diet is an important risk factor for dental caries, its cariogenic influence can be decreased using fluoride tooth paste [8, 11], having a good oral hygiene and balanced diet, and decreasing the consumption of high-sugar snacks between the main meals [12]. Harmful dietary habits and low tooth brushing frequency are both associated with caries experience [6, 13].

Although the prevalence of dental caries has significantly decreased in the industrialised countries during past decades, this trend has shown signs of reversing [14], particularly in terms of childhood caries [15]. A previous study from Finland also suggests a stagnation in the improvement of the cariological status among young males [16].

In Finland, military service is obligatory for men, and voluntary for women, under 28 years old, except if they have a physical or mental disability preventing the service [17]. Because 80% of men in each age cohort complete their service, the mandatory military service has provided an excellent opportunity in the past decades for epidemiological studies among young men.

Our hypothesis was that most young Finnish men snack daily and snacking is associated with both harmful health behaviours such as less frequent tooth brushing, smoking and snuffing, as well as favourable behaviours like high physical exercise activity. Another hypothesis was that the daily snacking is associated with both caries experience and restorative treatment need. The aim of this study was to investigate the prevalence of frequent snacking which is associated with both general and oral health.

## Methods

The calibration procedure, study protocol, study population, and questionnaire have been described in detail in previously published articles [16, 18, 19]. The study population comprised Finnish male and female conscripts born in 1990, 1991, and 1992 (mean age 19.6 years). A total of 8545 conscripts were requested to answer a computer-assisted questionnaire. Due to limited time, not everyone had an opportunity to answer the questionnaire [19]. In the questionnaire the response rate varied slightly between the questions (range from 8537 to 8545).

The following questions were used in the analyses: *During the past 6* *months, how often have you: Exercised or done sports?*/*Been to the movies, theatre, or sports events or played music or read?*/*Spent time with friends, played with friends?*/*Been to bars or pubs?*/*Watched TV or played computer games?*/*Been outdoors in the nature or gone fishing or hunting?*/*Done handicrafts (woodwork, metal work, painting)?*/*Done motor sports (car, motor cycle)?*); *How often do you eat/drink the following snacks: sweets*/*chocolate*/*crisps*/*fizzy drinks*/*energy drinks*/*sports drinks?*; *How often do you consume snack products in the following situations: commuting to school/work, at school/work, commuting from school/work to home, at home, playing computer/other devices, watching television/videos/films, reading books/magazines, spending time with friends, exercising, doing other activities?*; *How often do you use xylitol chewing gum?*; Answers for previous questions contained three options: *Never or hardly ever/Every day or almost every day/Occasionally during the week*. In addition, following questions were asked: *What is your education?* (comprehensive school/vocational school/university of applied sciences/vocational school and matriculation exam or upper secondary school/matriculation exam or upper secondary school/college or university/other); *How often do you brush your teeth?* (never or hardly ever/occasionally/every day). For those who reported brushing their teeth every day, an additional question was asked: *How many times a day do you brush your teeth?* (once a day/twice a day/more often than twice a day); *Have you received individual diet counselling?* (yes/no). The final three questions were: *Do you smoke?* (no/1–5 cigarettes daily/10–20 cigarettes daily/> 20 cigarettes daily); *Do you use snuff?*; *How often have you exercised during the past 6* *months?*. For the questions concerning the use of snuff and physical exercise the options were: *Never or hardly ever/Every day or almost every day/Occasionally.*

Clinical information was collected from the outcome of oral health screenings carried out on 13,564 Finnish male conscripts who entered military service in 2011. Because of the low number of female conscripts (*n* = 255), females were excluded from the final analyses. The oral health screening was performed as a part of the obligatory general health inspection during the conscripts’ first 2 weeks in the military service. There were no refusals to participate, as the clinical examination was obligatory. A representative sample of the 2011 batch of conscripts was achieved by examining all the conscripts in a total of 15 garrisons and every fifth conscript in the alphabetical order in the five largest garrisons. The dentists were trained and calibrated in two full-day sessions in November 2010 and June 2011. The inter-examiner and intra-examiner agreement regarding the teeth treatment need were determined for both sessions separately [16]. Caries experience was indicated by the mean DMFT (number of decayed, filled and missing teeth due to dental caries). The DT value represented the need for restorative treatment (both primary and secondary caries lesions). Third molars were excluded from all the analyses.

For statistical regression modelling, the DT value was used as the response variable. It was dichotomised as follows: DT = 0 or DT > 0. To enable analysing the subjects’ snacking habits, the responses to the questions concerning snacking (*How often do you eat/drink the following snacks: sweets*/*chocolate*/*crisps*/*fizzy drinks*/*energy drinks*/*sports drinks?*) were collated and categorised as follows: those who consumed at least one snack product every day or almost every day and those who did not report regular snacking. The confounding variables were categorised as follows: *non*-*smokers* and *those smoking at least one cigarette daily* and *non*-*snuffers* and *those using snuff at least occasionally during the week*. Those who use xylitol chewing gum every day or almost every day and those who did not report regular use. The responses to the two questions dealing with tooth brushing were combined and categorised in two different ways: first, they were categorised into those brushing their teeth *less often than once a day*, *once a day* or *twice or more often per day*, and then to *those brushing their teeth twice or more often per day* and *the rest*. The education level was dichotomised into *vocational school* and *the rest*. The daily exercise activity was dichotomised into *those exercising daily or almost every day* and *the rest*, and individual health promotion was dichotomised as *yes* or *no*.

Cross-tabulation was used to analyse the association between snacking habits and other health behaviours such as exercise activity, tooth brushing frequency, smoking or snuffing, individual health promotion (*Have you received individual diet counselling?* yes/no), education level, and language of the home municipality. Pearson’s Chi square test was used to study the statistical significance between the groups. Statistical significance was determined at *p* value < 0.05. The binary logistic regression analysis (adjusted, OR, 95% CI) was used for analysing the association between restorative treatment need (DT = 0, DT > 0) and snacking habits and other health behaviours. The mean (SD) DT and DMFT values were calculated in association with the dichotomised frequency of daily snacking. All analyses were executed using the SPSS software (versions 23.0, SPSS, Inc., Chicago, IL) and R software (version 3.1.2. A language and environment for statistical computing; R Foundation for Statistical Computing, Vienna, Austria, URL; http://www.R-project.org).

## Results

Almost one-third (30.0%) of the study group consumed snack products daily. Of these, 63.8% consumed one snack product daily whereas 22.8% consumed two snack products. Of those who reported daily snacking, 73.3% consumed fizzy drinks and 28.8% consumed energy drinks. The respective figures for the entire study population were 22.0% and 8.7%. Among those who reported daily snacking, tooth brushing twice or more often per day was not as common (46.5%) as among the rest of the study population (*n* = 3317, 55.5%) (*p* < 0.001) (Table 1a). The educational background of the vocational school was more common among the daily snackers (58.0%) compared to those who did not snack daily (52.0%) (*p* < 0.001). There was no difference in the conscripts’ daily physical exercise activity between those who consumed snack products daily and those who did not. Approximately 40% of those snacking daily also exercised daily, while the respective figure among the non-snackers was 40.6% (Table 1b).

**Table 1:** Cross tabulation and Pearson *χ*^2^-tests on association between snacking, brushing, and exercise activity among participants

Consumption of snacks %			
Teeth brushing	No snack products	At least one snack product daily	*p* value
(a) *n* = 8 537			
Less often than once a day	9.3% (555)	12.0% (311)	
Once a day	35.2% (2101)	41.4% (1062)	
Twice or more per day	55.5% (3317)	46.5% (1191)	< 0.001
Total	100% (5973)	100% (2564)	

Consumption of snacks %			
Exercise activity	No snack products	At least one snack product daily	*p* value
(b) *n* = 8545			
Daily or almost every day	40.6% (2426)	40.4% (1036)	
Occasionally	50.5% (3020)	46.0% (1181)	
Never or hardly ever	8.9% (532)	13.6% (350)	< 0.001
Total	100% (5978)	100% (2567)	

Smoking at least ten cigarettes per day was statistically significantly more common among those snacking at least one snack product daily compared to the subjects who did not report daily snacking (33.4% vs 23.6%; *p* < 0.001). The subjects who reported daily snacking also reported daily snuffing more often (11.1%) than those who did not snack (8.1%) (*p* < 0.001).

**Fig. 1:**
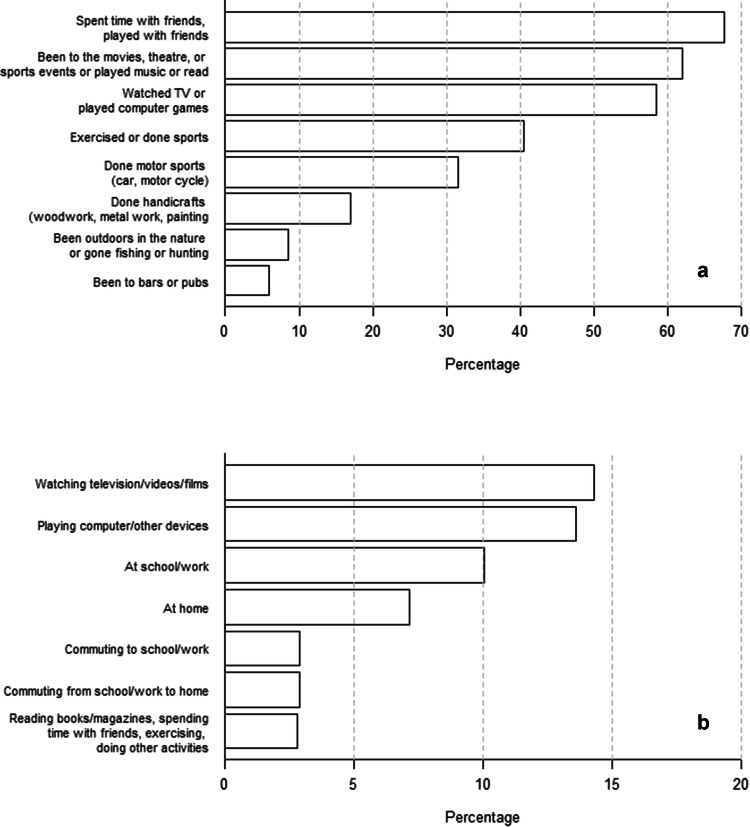
**a** Distribution of activities which conscripts have done every day or almost every day during the past 6 months. **b** Prevalence of daily snacking during the activities which conscripts have done during the past 6 months

More than half of the study population had played computer games or watched television daily during the past 6 months. However, spending time with friends and going to movies, theatre or sports events, playing music or reading were even more common activities (Fig. 1a). Snack products were most commonly consumed while watching a film at the cinema (Fig. 1b). The majority (89.5%) of the study population brushed their teeth every day or almost every day, and 73.5% of them used xylitol chewing gum. Only about one-tenth (12.2%) had received an individual counselling on dietary issues.

The prevalence of restorative treatment need was statistically significantly (*p* < 0.001) higher among the conscripts who consumed snacks regularly (52.5%) compared to those who did not (45.0%) (Table 2). Consumption of xylitol chewing gum daily, again, was less common among those with restorative treatment need (18.5%) than the rest (22.9%) (*p* < 0.001).

**Table 2:** Distribution of restorative treatment need and mean DT/DMFT of participants dichotomised according to snacking habits

Group	DT	DMFT	Restorative treatment need of participants *n* (%)	
	Mean (SD)	Mean (SD)	Yes	No
At least one snack product daily or almost every day (29.8%)	1.83 (3.00)	4.90 (4.62)	1347 (52.5%)	1220 (47.5%)
No snack products daily (70.2%)	1.35 (2.38)	3.99 (4.05)	2 689 (45.0%)	3 289 (55.0%)
*p* value	< 0.001	< 0.001		< 0.001

Tests used: independent sample *t* tests and cross-tabulation and Pearson’s Chi square tests (*n* = 8545)

According to the logistic regression analysis, daily consumption of snacks and smoking significantly increased the odds for restorative treatment need, while brushing teeth twice a day and a higher education level decreased the odds (Table 3).

**Table 3:** Adjusted logistic regression analysis on association between explanatory variables and restorative treatment need (DT > 0)

*Snacking*		
No snack products	1	
At least one snack product daily	1.19	(1.07, 1.32)
*Tooth brushing*		
Less often than twice a day	1	
Twice or more often per day	0.80	(0.73, 0.88)
*Education*		
Vocational school	1	
Matriculation exam or upper secondary school or other	0.56	(0.51, 0.62)
*Individual health promotion on healthy diet*		
Yes	1	
No	0.90	(0.78, 1.03)
*Smoking*		
No	1	
Yes	1.63	(1.47, 1.81)
*Snuffing*		
No	1	
Yes	1.02	(0.91, 1.15)
*Exercise activity*		
Other	1	
Daily	1.09	(0.99, 1.20)
*Xylitol chewing gum*		
No	1	
Yes	0.89	(0.76, 1.03)

## Discussion

Finnish young men seem to snack frequently, for instance, while playing computer games. Fizzy and energy drinks are common snack products consumed. As hypothesized, snacking is associated with both smoking and snuffing, but against our hypothesis not with physical activity. Regular snacking is associated with restorative treatment need and history.

In a Swedish study by Bruno-Ambrosius et al. [20], approximately 20% of the subjects consumed sweet snacks daily or almost every day, which is in line with our results. Some other studies have also received similar results concerning snacking frequency [3, 21]. In our study, daily consumption of sweets was reported by almost 7%, which is somewhat higher than in a recent Finnish school oral health survey [22], when 5% of vocational school-aged boys reported consuming sweets or chocolate daily. Surprisingly, although, almost a third of the study population consumed snacks daily and a half had restorative treatment need [16], only 10% of the subjects had received dietary counselling.

Snacking seems to be associated with other harmful health behaviours such as smoking and snuffing. In addition, those who report brushing their teeth less frequently snack more often. In the present study, there was no association between snacking and high activity of physical exercise, which is contradictory with results from a study on young athletes [23]. However, there was a light association between high physical exercise activity and restorative treatment need.

In the present study, approximately one-tenth of the study population reported daily consumption of energy drinks. Similar results were found in a recent Finnish study [22], in which 8% of men in vocational school reported consuming energy drinks daily or almost daily. The respective figure for men in upper secondary school (i.e. the Finnish equivalent of American high school) was 2%. This supports our finding that the lower educational background is associated with snacking. The reason for that is not known. Perhaps good cognitive skills due to a higher educational background can also protect against unhealthy behaviours such as snacking, but also with snuffing and smoking [18].

According to a recent Swedish study [24], regular consumption of fizzy drinks is associated with poor oral health and an unhealthy lifestyle per se. In this study, the DMFT and DT values were significantly higher among the conscripts who consumed at least one snack product daily and the tooth brushing frequency was low. Our results concerning the association between caries indices and associated factors are supported by existing literature. Akarslan et al. [6] have found a similar correlation between the DMFT index and snacking frequency. However, contrary to our study, they found no significant association between snacking habits and tooth brushing frequency [6]. Sabbah et al. [25] have found an association between the DMFT and DT values and lower education level, which was true here as well. This outcome is also supported by Nguyen et al. [26], who reported that the dental caries is negatively associated with education and healthy diet. Previous studies have found differences in the oral health behaviour [6, 23], caries prevalence [25], and associated factors [26] between males and females, which could not be studied here due to the limited number of females in the study population.

Dental caries is a significant public health problem among Finnish young men, as almost half of the young men have at least one tooth needing restorative treatment and only around 20% are completely caries free [16]. One reason may be that this age group have lived their teens in time when snacking and consumption of fizzy drinks have increasingly replaced regular meals [27]. Bernabe et al. [8] have reported that the frequency of sugar consumption and the amount of consumed sugars are associated with dental caries in Finnish adults, which supports our results. Sheiham et al. [7] have also reported a similar link between sugar consumption and caries.

An interesting and alarming finding of this study is that only a small proportion of the conscripts had received individual dietary counselling. Reasons for this can only be speculated. In Finland, not everyone routinely visits dental hygienists, who are specialists in health promotion in the field of public dental care. It is also possible that the dentists are not used to give diet counselling or even referring the patient to oral hygienist for this. The dentists may even consider an individual dietary counselling outside the scope of their job description. They may not be aware of new methods for oral health promotion like motivational interviewing which is an effective way to motivate adolescents to improve their poor dietary and oral hygiene behaviours [28]. Health promotion may also be challenging. According to a study by Stokes et al. [29], even if young people are aware of the risk factors threatening their oral health, they may not consider changing their health behaviour as important. Perhaps, these young people live more in the present without thinking about the potential consequences of their choices and behaviours in future.

The strength of this study is large and representative study population was born in the early 1990s in Finland. Investigating the variety of different background variables associated with snacking and oral health can also be considered advantageous for this study. However, not everyone had a chance to answer the computer-based questionnaire due to time constraints in the health examinations of the defence forces. Therefore, the response rate was lower than the number of participants in the clinical screening. The number of responses also varied slightly between the questions. These can be considered as a shortcoming of this study. The study was performed during the conscripts’ first two weeks of military service. Therefore, it can be assumed that the military service had not yet influenced the conscripts’ health behaviour and they could also recall behaviours in civil life. It would have been valuable if the questions about the snacking habits had been more precise, such as questions about the frequency of snacking per day. Another weakness was the limited number of female conscripts, which meant that all the results concerning females had to be excluded from the analyses. A cross-sectional study can only provide a snapshot of conscripts’ health habits and association, which makes it impossible to draw cause–effect conclusions about the association between snacking and dental caries.

It can be concluded that daily snacking is common and associates with other harmful health behaviours and restorative treatment need among Finnish conscripts. Individual dietary counselling should be routinely offered as part of dental treatment and especially caries controlling in dental clinics. This could be beneficial for general health as well.
